# Mechanical and Thermomechanical Performance Evaluation of CFRP Sandwich Structures with Additively Manufactured Composite Cores

**DOI:** 10.3390/polym18202510

**Published:** 2026-10-14

**Authors:** Alexandru Ciubotariu, Teodor-Adrian Badea, Madalina Mustareata, Raluca Maier, Andrei Timonia, Vlad Buga, Laurentiu Petre, Ciprian Ionuț Morăraș, Viorel Goanță

**Affiliations:** 1 Composite Materials Laboratory for Aeronautical Field, Romanian Research & Development Institute for Gas Turbines—COMOTI, 220D Iuliu Maniu Av., 061126 Bucharest, Romaniateodor.badea@comoti.ro (T.-A.B.); raluca.maier@comoti.ro (R.M.); andrei.timonia@comoti.ro (A.T.); vlad.buga@comoti.ro (V.B.); laurentiu.petre@comoti.ro (L.P.); 2 Mechanical Engineering, Mechatronics and Robotics Department, Mechanical Engineering Faculty, “Gheorghe Asachi” Technical University of Iasi, 700050 Iasi, Romania; ciprian-ionut.moraras@academic.tuiasi.ro (C.I.M.); viorel.goanta@academic.tuiasi.ro (V.G.)

**Keywords:** sandwich structure, CFRP composite, 3D-printed core, flatwise compression, flexural tests, dynamic mechanical analysis (DMA)

## Abstract

This study experimentally evaluates how various 3D-printed gyroid core materials influence the mechanical and thermo-mechanical performance of lightweight carbon-fiber-reinforced polymer (CFRP) sandwich structures. Three gyroid core configurations were considered: short-carbon-fiber-reinforced polyphthalamide (PPA-CF), continuous-carbon-fiber-reinforced nylon (ONYX-R), and nylon short-carbon-fiber-reinforced (ONYX), all combined with similar 0/90° carbon-fiber-reinforced polymer composite (CFRP) face sheets. The sandwich specimens were manufactured using a one-step co-curing process and characterized by flatwise compression, three-point bending, and dynamic mechanical analysis (DMA). The PPA-CF-CFRP sandwich configuration exhibited the highest flexural performance, with a maximum flexural stress of 148–156 MPa, approximately 39% higher than that of ONYX-R-CFRP, and the highest mean compression modulus of approximately 87 MPa. However, ONYX-R-CFRP achieved the highest mean compressive strength of 12.84 MPa, compared with 11.85 MPa for PPA-CF-CFRP. These findings indicate that the mechanical response depends on the loading mode and is influenced by the combined effects of the thermoplastic matrix, carbon-fiber reinforcement architecture, and the resulting structural characteristics of the printed gyroid cores. DMA revealed that the PPA-CF-CFRP sandwich achieved a storage modulus of ~9 GPa at 30 °C, outperforming the ONYX-R-CFRP configuration by approximately 70%. Conversely, ONYX-R-CFRP exhibited the highest damping capacity, with a maximum tan δ of approximately 0.16, about 60% higher than the PPA-CF-CFRP configuration. The ONYX-CFRP sandwich configuration displayed the lowest mechanical and thermo-mechanical performance, demonstrating that the stiffness, deformation capacity, damping response, and temperature-dependent mechanical behavior of these sandwich structures can be tailored through the selection of the additively manufactured core configuration.

## 1. Introduction

Sandwich structures are widely used in aerospace, transportation, marine, and energy applications due to their ability to provide high specific stiffness and strength while maintaining a low structural weight. Compared with monolithic constructions of similar mass, they provide potentially higher bending stiffness and energy absorption by combining stiff skins with a lightweight core, making them a preferred solution for weight-critical engineering systems [1,2]. Conventional sandwich structures typically rely on metallic honeycomb, polymer foam, or balsa wood cores; however, these conventional architectures offer less freedom to vary the internal geometry than additively manufactured cores [1,2].

Recent advances in additive manufacturing (AM) have enabled the fabrication of architected cellular materials with designed cellular geometries, adding geometric design variables to material selection. Mechanical properties can be tailored through unit-cell topology and relative density, making 3D-printed lattice structures attractive alternatives to conventional sandwich cores because of their adjustable stiffness, strength, and energy absorption capabilities [2,3]. Among AM technologies, fused filament fabrication (FFF) is one of the most widely used methods for producing polymer lattice structures [4], while Continuous Fiber Fabrication (CFF) further enhances mechanical performance by embedding continuous reinforcing fibers within the thermoplastic matrix. Previous studies have demonstrated that fiber architecture strongly influences the compression response and failure mechanisms of printed composite structures, highlighting the potential of continuous-fiber AM for high-performance sandwich cores [5,6].

Early investigations confirmed that conventional sandwich cores can be successfully replaced by additively manufactured lattice architectures, with core topology playing a key role in stiffness, strength, bending response, and failure mechanisms [7,8]. Among the available lattice families, triply periodic minimal surface (TPMS) structures, particularly the gyroid topology, have attracted considerable attention because their continuous, self-supporting geometry can provide interconnected load paths, although specific properties depend on density, defects, and loading direction [9,10]. Bio-inspired structural concepts have therefore emerged as an important strategy for improving lightweight structural efficiency [11,12]. Owing to its complex three-dimensional geometry, the gyroid is impractical to manufacture using conventional techniques but can be readily fabricated through additive manufacturing [13].

Several studies have demonstrated the effectiveness of gyroid-based sandwich structures. Pelanconi and Ortona [14] and Ayrilmis et al. [15] showed that both lattice geometry and face-sheet configuration significantly influence the stiffness and mechanical performance of gyroid-core sandwich panels. Additional experimental investigations confirmed the flexural performance and weight efficiency of gyroid lattice cores [16,17], while modified architectures incorporating continuous outer layers were shown to improve the skin–core interface by increasing the bonding area and reducing stress concentrations [18]. Likewise, hybrid sandwich structures combining CFRP prepreg skins with additively manufactured cores have demonstrated high structural efficiency and manufacturing feasibility for lightweight applications [7,19].

Thermoplastic composites are particularly attractive for additive manufacturing because they can be processed through melting and solidification without requiring a separate chemical curing reaction. Compared with conventional thermosetting composites, they offer advantages in terms of processing flexibility, potential recyclability, and the fabrication of complex geometries through layer-by-layer deposition. However, their performance remains sensitive to processing temperature, interlayer bonding, porosity, reinforcement distribution, and fiber–matrix interfacial characteristics [20]. Carbon-fiber-reinforced polyamides are suitable for mechanically loaded printed components, while higher-performance thermoplastic matrices, such as polyphthalamide, offer potential advantages in stiffness and elevated-temperature applications. These characteristics make reinforced thermoplastics promising candidates for customized lattice cores, although their structural performance must be evaluated at the sandwich level rather than inferred solely from the properties of the constituent materials.

Among thermoplastic materials suitable for additive manufacturing, ONYX is a short-carbon-fiber-reinforced nylon thermoplastic, while ONYX-R additionally incorporates continuous carbon-fiber reinforcement, providing increased stiffness and strength compared with unreinforced thermoplastic systems [21]. Short Carbon-fiber-reinforced polyphthalamide (PPA-CF), on the other hand, offers high stiffness, load-bearing capability, and improved thermo-mechanical performance, making it a promising material for structurally loaded additively manufactured components [22,23]. Nevertheless, the performance of an AM sandwich core cannot be assessed solely from its constituent material properties, as the resulting structural response is also governed by the core architecture, skin–core interaction, and overall mass. Additively manufactured lattice core sandwich structures have emerged as a high-interest research domain, driven by their potential to deliver superior thermo-mechanical performance relative to conventional sandwich panels. The authors revealed in prior research [24] the mechanical and thermo-mechanical behavior of conventional Coreflex and Nomex-core sandwich structures. Although previous studies have investigated the mechanical performance of additively manufactured gyroid cores and CFRP-faced sandwich structures, comparative experimental evidence remains limited regarding the combined influence of thermoplastic matrix selection and carbon-fiber reinforcement architecture on the static and thermomechanical response of complete sandwich panels. In particular, the extent to which short-carbon-fiber-reinforced PPA and nylon-based cores with and without additional continuous carbon-fiber reinforcement differ under flatwise compression, three-point bending, and dynamic mechanical loading has not been clearly established under a common experimental framework. The present study addresses this question by comparing three manufactured gyroid-core configurations with the same nominal topology, infill setting, and CFRP face-sheet arrangement. Since the manufacturing parameters also differ among configurations, the comparison is intended to assess their resulting structural performance rather than isolate the intrinsic contribution of each constituent material. The three core-material configurations were selected to enable a systematic comparison of different thermoplastic matrices and carbon-fiber reinforcement architectures within the same gyroid lattice topology. ONYX was selected as a short-carbon-fiber-reinforced nylon reference, while ONYX-R was selected to investigate the effect of introducing continuous carbon-fiber reinforcement into the same ONYX-based matrix. PPA-CF was included as a short-carbon-fiber-reinforced polyphthalamide system, providing a different thermoplastic matrix with high stiffness, load-bearing capability, and improved thermo-mechanical stability. This combination therefore allowed the effects of matrix type and fiber-reinforcement architecture on the mechanical and thermo-mechanical response of the sandwich structures to be evaluated. To minimize the influence of geometry, all three core configurations were manufactured using the same TPMS gyroid topology, approximately 6 mm overall thickness, and 28% infill.

Therefore, the objective of the present study is to evaluate the response of three printed gyroid-core configurations on the mechanical, thermo-mechanical, and weight characteristics of sandwich structures. By leveraging the inherent benefits of additive manufacturing, such as geometric customization flexibility, mold-free fabrication of complex topologies, and minimized material waste, this work aims to provide a framework for further structural optimization. The results provide an experimental basis for selecting additively manufactured gyroid-core configurations for lightweight sandwich structures subjected to compressive, flexural, and thermomechanical loading. Potential applications include weight-sensitive structural components in aerospace and transportation; however, their suitability for specific operating environments requires further validation, including impact, fatigue, and environmental testing.

## 2. Materials and Methods

### 2.1. Materials

The selection of the core materials was driven by the increasing demand for lightweight sandwich structures with enhanced mechanical performance, improved design flexibility, and simplified manufacturing for aerospace and other weight-sensitive engineering applications. Unlike conventional honeycomb cores, additively manufactured lattice structures offer the possibility of tailoring the mechanical response through the selection of both the material and the internal architecture while enabling the fabrication of complex geometries that are difficult or impossible to manufacture using traditional techniques.

Therefore, three different gyroid lattice core configurations were investigated in the present study: short-carbon-fiber-reinforced polyphthalamide (PPA-CF), continuous-carbon-fiber-reinforced nylon ONYX (ONYX-R), and ONYX (ONYX, short-carbon-fiber-reinforced nylon). All lattice cores had an overall thickness of ~6 mm and near-identical gyroid geometries to ensure that the influence of the investigated core configuration on the structural performance could be directly evaluated.

ONYX is a nylon (polyamide 6) composite reinforced with 10% to 20% short carbon fibers by volume, as reported in [25], while image analysis conducted by Sauer [26] revealed a short fiber content of approximately 9% within the matrix phase.

ONYX-R consists of an ONYX matrix reinforced with continuous carbon fibers. The continuous-fiber content is controlled by the fiber-infill configuration during printing.

PPA-CF is a polyphthalamide (PPA) thermoplastic composite reinforced with 25 wt.% short carbon fibers. The PPA-CF cores were manufactured using a 1.75 mm filament diameter, supplied by Raise3D Hyper Core.

The continuous-carbon-fiber-reinforced ONYX was selected because of its high stiffness and strength, whereas PPA-CF was chosen for its excellent thermo-mechanical stability and load-bearing capability.

**Figure 1:**
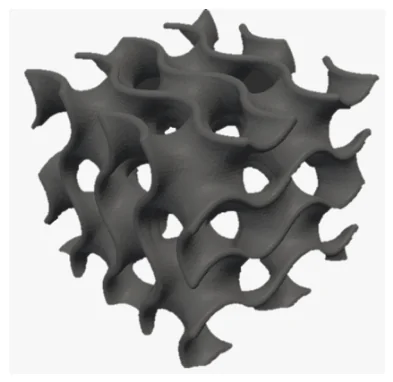
Section of gyroid lattice.

Furthermore, the architecture of the printed core represented one of the main variables investigated in this study. All sandwich configurations employed the same triply periodic minimal surface (TPMS) gyroid geometry (28% infill), providing a common nominal lattice topology for comparing the manufactured core configurations, while acknowledging differences in material composition, reinforcement architecture, and processing parameters. The 28% value refers to the nominal infill setting defined in the slicing software and should not be interpreted as an experimentally measured relative density of the manufactured cores. Figure 1 illustrates the geometries of the investigated gyroid lattice cores.

For the fabrication of the sandwich face sheets, HexPly M49 carbon-fiber-reinforced epoxy prepreg supplied by Hexcel was used, with a nominal resin content of 42 wt.%. The prepreg was chosen due to its high specific stiffness, excellent mechanical properties, and widespread use in lightweight aerospace structures. Since all the sandwich configurations investigated employed identical CFRP face sheets, the influence of the investigated core configuration on the global mechanical behavior could be directly assessed. The sandwich panels were manufactured through a one-step co-curing process, in which the prepreg face sheets were directly bonded to the printed lattice core during curing without the use of an additional adhesive film. This approach simplifies the manufacturing process while ensuring a continuous bond between the CFRP skins and the thermoplastic lattice core. The printing files were prepared using the dedicated slicing software associated with each additive manufacturing system. The PPA-CF cores were processed using Prusa Slicer (version 2.9.4) and printed with Prusa CoreOneL (Prusa Research a.s., Prague, Czech Republic), whereas the ONYX and ONYX-R cores were prepared using Markforged Eigersoftware (https://markforged.com/software, Accessed 16 July 2026) and printed with Marforged X7 (Markforged, Watertown, MA, USA). The slicing stage allowed the definition of the printing strategy, including layer height, wall generation, infill strategy, printing orientation, and fiber reinforcement. For the ONYX-R configuration, continuous carbon fiber reinforcement was introduced using the Markforged Eiger softwareby defining the number of fiber layers, fiber rings, and reinforcement orientation according to the desired mechanical performance. In contrast, the PPA-CF and ONYX configurations were manufactured without continuous fiber reinforcement while maintaining identical geometrical parameters. The main printing parameters adopted for each material are summarized in Table 1. The nominal continuous carbon-fiber volume fractions reported for the ONYX-R cores were calculated by dividing the volume of continuous carbon-fiber reinforcement by the total external volume of the corresponding printed core and multiplying by 100. The calculated values were approximately 8.0%, 9.3%, and 7.4% for the compression, three-point bending, and DMA specimens, respectively. These differences reflect the specimen-specific geometries and reinforcement layouts. The calculated fractions were not independently verified through microscopic or destructive characterization.

The mass of each additively manufactured gyroid core was experimentally determined using a weighing scale prior to the application of the CFRP face sheets. The measured core masses for the DMA, flatwise compression, and three-point bending specimens are presented in Table 2. Although all configurations were manufactured using the same nominal gyroid infill percentage of 28%, differences in material composition, reinforcement architecture, and printing parameters resulted in different core masses. The nominal extrusion track width was approximately 0.45 mm for the Prusa-printed PPA-CF cores and 0.40 mm for the Markforged-printed ONYX and ONYX-R cores, based on reference printing parameters for 0.4 mm nozzles. These values are provided as nominal manufacturing characteristics and should not be interpreted as experimentally measured gyroid wall thicknesses, which were not independently determined. Although the investigated cores were manufactured using the same nominal gyroid infill percentage (28%) and comparable overall dimensions, the printing equipment, layer heights, top and bottom layers, and reinforcement strategies differed between configurations, as detailed in Table 1. The measured mechanical and thermomechanical responses should therefore be interpreted as the combined influence of material composition, reinforcement architecture, and manufacturing parameters. The apparent densities of the additively manufactured gyroid cores were calculated from their experimentally measured masses and nominal external dimensions, prior to CFRP face-sheet application. The resulting values ranged from 0.477 to 0.590 g/cm^3^ across the investigated configurations and specimen geometries. These values characterize the manufactured cores and should be distinguished from the apparent densities of the complete CFRP sandwich specimens.

**Figure 2:**
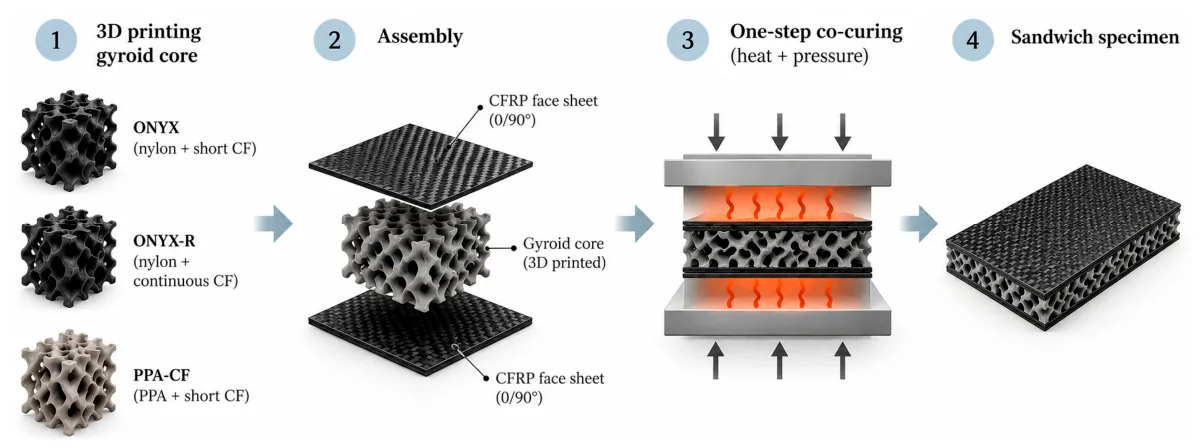
Sample manufacturing process.

Following the fabrication of the gyroid lattice cores, the sandwich specimens were assembled by manually applying CFRP prepreg face sheets to both sides of each printed core. The prepreg plies were previously cut to the required dimensions using a CNC Eastman Eagle S125-60 × 8 cutting system (Eastman Machine Company, Buffalo, NY, USA), according to the geometry of each mechanical test specimen. Each sandwich structure consisted of a 3D-printed gyroid core positioned between two CFRP prepreg plies on each side, with a [0/90]/core/[90/0] lay-up, resulting in a symmetric configuration with a total of four prepreg plies per specimen. The plies were manually aligned to ensure complete coverage of the printed core and proper positioning of the composite face sheets. Prior to the application of the CFRP prepreg face sheets, the surfaces of all additively manufactured gyroid cores were mechanically sanded and subsequently cleaned to remove surface irregularities and residual particles. This preparation procedure was applied to all three core configurations before the CFRP prepreg layers were placed directly onto the thermoplastic cores, without the use of an additional adhesive film. The sandwich assemblies were positioned on a metallic mold plate previously coated with a release agent and subsequently debulked under a vacuum pressure of −0.9 bar to promote proper consolidation of the prepreg layers. After completing the lay-up, the assemblies were prepared for vacuum bagging by placing a release film and breather layer over the laminate, followed by sealing within a vacuum bag. Two vacuum lines were used, one for vacuum applications and the second for monitoring the vacuum level throughout the curing process. The vacuum-bagged assemblies were cured in an oven following the HexPly M49 prepreg recommended cycle under a constant −0.9 bar vacuum, ensuring laminate consolidation, promoting intimate contact between the CFRP face sheets and the printed gyroid core, and minimizing void formation. The resulting sandwich architecture and bagging configuration are shown in Figure 2.

The CFRP sandwich structures were manufactured using a one-stage curing cycle, following the recommended curing conditions for the HexPly M49 prepreg system: the temperature was increased at a controlled heating rate of 3 °C/min to 120 °C and maintained at this temperature for 120 min. The structures were subsequently cooled to 60 °C at a rate of 3 °C/min before being removed from the metallic mold. After curing, the manufactured panels were carefully removed from the mold and trimmed to the required dimensions using a fine grinding wheel disk cutter. The cutting procedure was performed with minimal material removal to avoid damaging the CFRP skins or the core structures. Three sandwich configurations were manufactured using the same CFRP face sheets and three different additively manufactured core materials: PPA-CF, ONYX-R, and ONYX.

### 2.2. Experimental Setup

#### 2.2.1. Compression Tests

The flatwise compressive behavior of the sandwich specimens was characterized using an Instron 8801(Instron, Norwood, MA, USA) universal testing system equipped with a 100 kN load capacity. Testing was carried out in accordance with ASTM C365 [27] at 23 ± 3 °C and 50 ± 5% relative humidity. A displacement-controlled loading regime was applied at a crosshead rate of 0.5 mm/min, and each test was continued up to specimen failure. The tested coupons had in-plane dimensions of 60 × 60 mm, corresponding to a loaded area of 3600 mm^2^, whereas the specimen thickness was defined by the overall thickness of each sandwich configuration. For every investigated configuration, three independently manufactured specimens were tested (*n* = 3) in order to assess the repeatability of the experimental results.

#### 2.2.2. Three-Point Bending Tests

**Figure 3:**
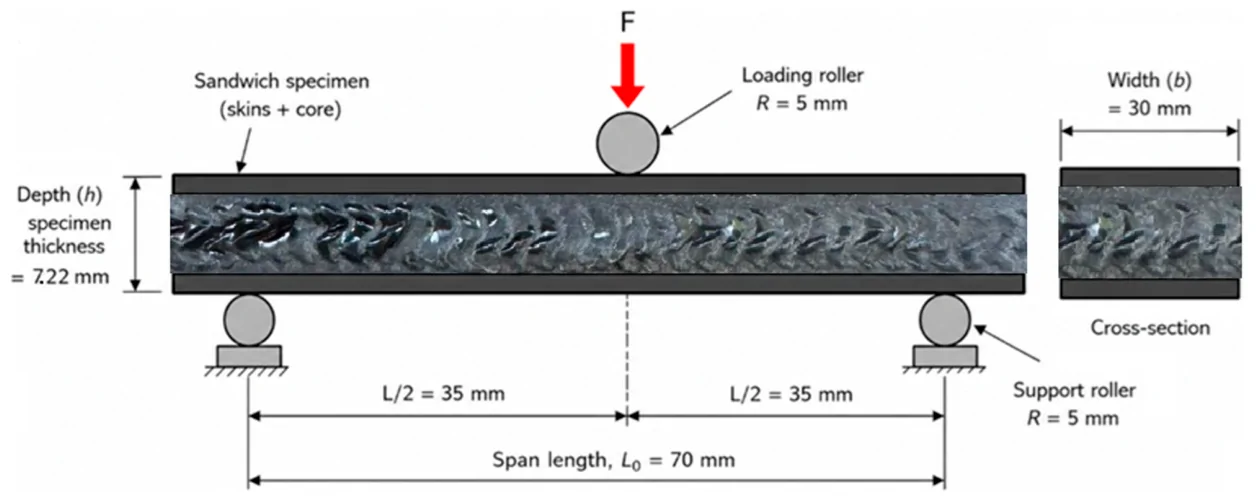
Schematic representation of the three-point bending test configuration according to ASTM C393/C393M [28].

The shear-related flexural response of the sandwich specimens was investigated by three-point bending in accordance with ASTM C393/C393M [28]. Experiments were performed using an Instron 34SC-5 universal testing machine (Instron, ITW—Illinois Tool Works, Norwood, MA, USA) fitted with a 5 kN load cell. Rectangular specimens measuring 120 mm in length and 30 mm in width were tested, while their thickness corresponded to the overall thickness of the respective sandwich configuration. The support span was set to 70 mm, and rollers with a radius of 5 mm were employed in the test fixture. All measurements were conducted under laboratory conditions of 23 ± 3 °C and 50 ± 5% relative humidity. Loading was applied at a crosshead displacement rate of 6 mm/min until either specimen failure occurred or the imposed deflection reached a value equal to the specimen thickness. Three independently manufactured specimens were tested for each sandwich configuration (*n* = 3). The corresponding test arrangement is illustrated in Figure 3.

#### 2.2.3. Dynamic Mechanical Analysis (DMA)

The temperature-dependent viscoelastic response of the sandwich configurations was assessed by dynamic mechanical analysis using a TA Instruments DMA 850 system (New Castle, DE, USA) operated in three-point bending mode. Measurements were carried out at an oscillation frequency of 1 Hz in accordance with ASTM D7028-07(2015) [29]. The specimens were subjected to a temperature sweep from room temperature to 140 °C using a heating rate of 5 ± 1 °C/min. According to the instrument specifications, the modulus accuracy is ±1%, while the isothermal temperature stability above 50 °C is better than ±0.1 °C. The reported resolution and sensitivity for tan δ are 1 × 10^−5^ and 1 × 10^−4^, respectively. Rectangular specimens with dimensions of 59 ± 1 mm × 12 ± 1 mm × 6.5 ± 0.5 mm were examined, with three replicate samples analyzed for each sandwich configuration (*n* = 3). Storage modulus, loss modulus, and damping factor (tan δ) were recorded as functions of temperature in order to evaluate the thermomechanical response and identify the apparent transition regions of the investigated structures.

## 3. Results

### 3.1. Compression Test Results

**Figure 4:**
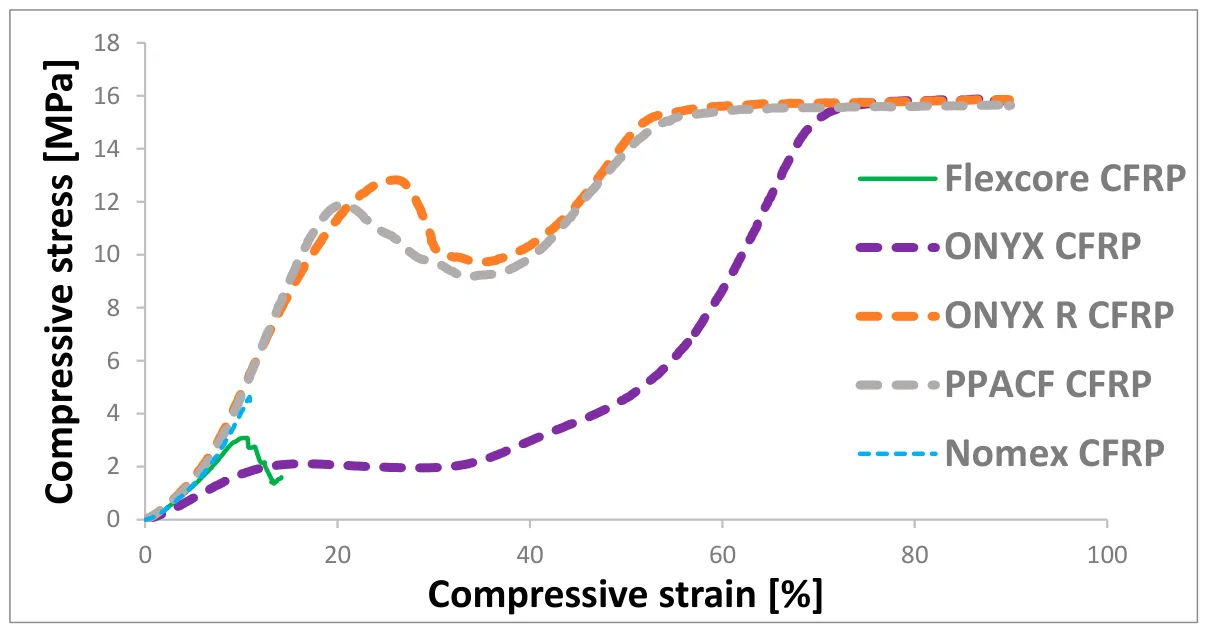
Mean flatwise compression stress–strain curves, including comparative data from [24].

In out-of-plane (flatwise) compression, the load is applied perpendicular to the sandwich panel faces, primarily loading the core in the through-thickness direction. The compression strength is primarily governed by the core material, its density, and geometry (in this case a gyroid structure). The high stiffness of the CFRP face sheets ensures efficient load distribution throughout the core, while the quality of the skin-to-core interface also significantly influences the overall compression response. The compression stress–strain curves obtained for all investigated sandwich configurations are presented in Figure 4, while the compression modulus, yield strength, compression strength, and corresponding averaged values are given in Table 3.

Additionally, the averaged curves for the CFRP-faced Flexcore and Nomex sandwich structures from our previous study [24] are included in Figure 4 for subsequent comparison in the Section 4.

It should be noted that the reference results reported in our previous study [24] were obtained by the same research team, using the same testing equipment, experimental procedures, and test conditions as those employed in the present study. Moreover, the specimens considered in both studies were manufactured and tested within the same experimental campaign, ensuring direct comparability of the resulting mechanical response and stress–strain curves.

### 3.2. Three-Point Bending Results

In a sandwich structure, the internal core separates the face sheets, increasing the cross-sectional moment of inertia and consequently enhancing the flexural properties of the structure. During three-point bending, the upper face sheet is subjected to compression, the lower face sheet to tension, while the core primarily withstands the resulting shear stresses. Therefore, the flexural response of the sandwich structure is strongly dependent on the mechanical properties of the core and its interaction with the composite face sheets.

**Figure 5:**
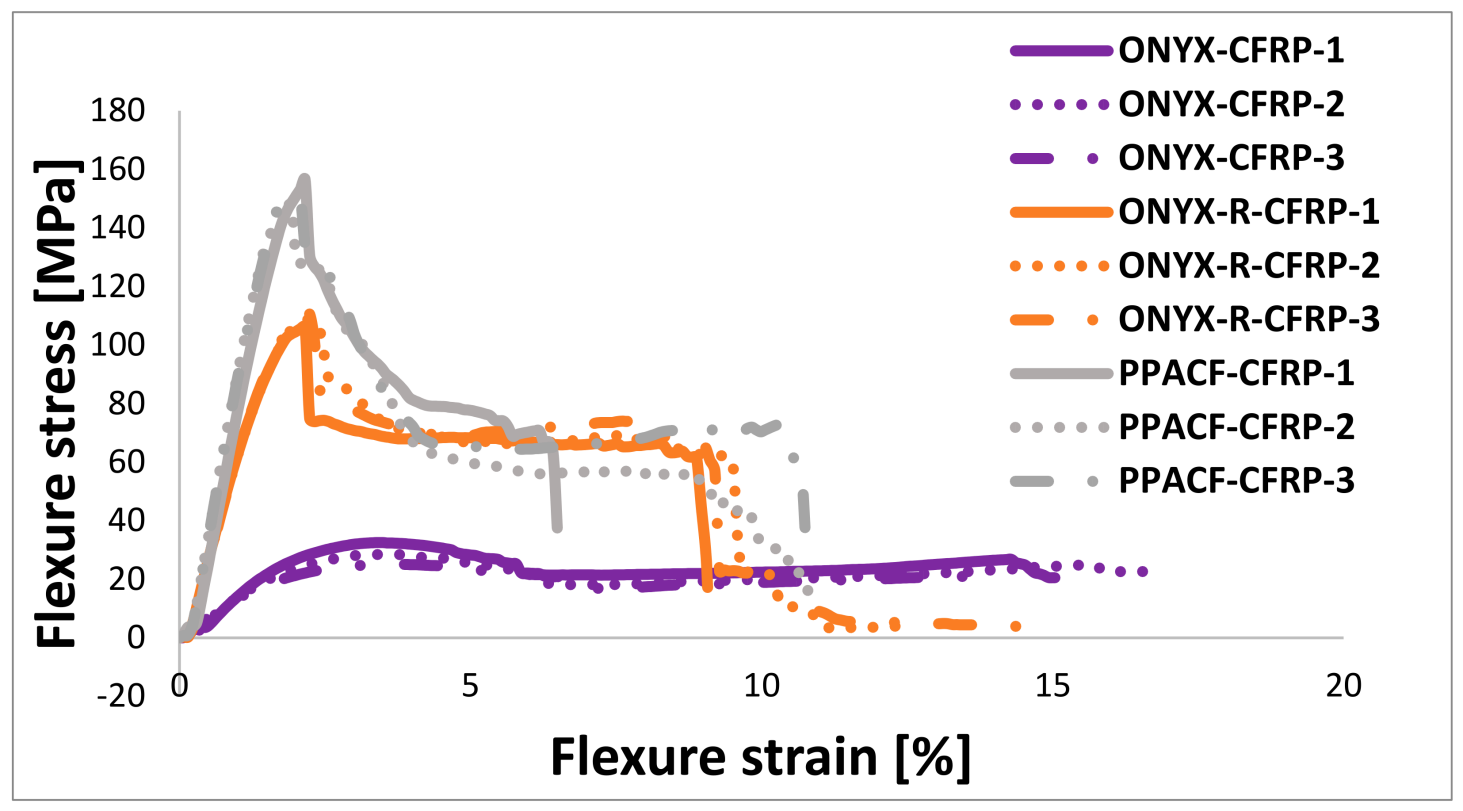
Reported three-point bending stress–strain curves for the sandwich configurations.

Figure 5 presents the flexural stress–strain curves obtained from all three-point bending tests. The investigated sandwich structures exhibited distinct mechanical responses depending on the core configuration. The ONYX-CFRP specimens showed the lowest flexural stress, reaching maximum values of approximately 25–32 MPa, while exhibiting the highest deformation capacity, with the curves extending to approximately 20–22% strain at break, as reported in Table 4. In contrast, the ONYX-R-CFRP configuration exhibited a substantially higher flexural strength, reaching approximately 106–111 MPa, followed by a gradual decrease in load-bearing capacity after the maximum stress was reached. The PPA-CF-CFRP configuration provided the highest flexural stress, reaching approximately 149–156 MPa. However, after reaching the maximum stress, a pronounced reduction in the flexural response was observed, indicating a more abrupt failure of the structure. The PPA-CF-CFRP specimens also exhibited a lower strain at failure compared with the ONYX-CFRP configuration. Overall, the results demonstrate that the core configuration has a pronounced influence on both the flexural strength and deformation behavior of the sandwich structures, with PPA-CF-CFRP providing the highest load-bearing capacity, while ONYX-CFRP exhibited the greatest deformation capability.

A substantial difference can be observed between the ONYX-R-CFRP and ONYX-CFRP configurations. This represents an increase of more than three times in the maximum flexural stress, accompanied by a reduction in deformation capacity. While the ONYX-CFRP specimens maintained a relatively stable response over a large strain range, the ONYX-R-CFRP specimens exhibited a distinct peak followed by a progressive decrease in load-bearing capacity. This behavior may be associated with differences in stiffness, reinforcement architecture, and manufacturing characteristics among the investigated gyroid-core configurations. In the ONYX-CFRP configuration, the comparatively compliant short-fiber-reinforced ONYX core allows greater deformation of the gyroid cell walls, resulting in higher global strain but limited stress transfer between the CFRP face sheets. The presence of continuous carbon-fiber reinforcement in ONYX-R is associated with a substantially higher flexural stress and lower deformation capacity compared with ONYX-CFRP. This behavior is consistent with a stiffer reinforced core architecture, although the individual contributions of fiber reinforcement, manufacturing parameters, and skin–core interaction were not independently quantified. PPA-CF-CFRP exhibits an even higher flexural response, indicating that the global behavior is not governed by fiber continuity alone, but by the combined effects of matrix properties, reinforcement distribution, cell-wall stiffness, and the interaction between the core and CFRP face sheets.

The PPA-CF-CFRP and ONYX-R-CFRP sandwich configurations displayed a substantial improvement in load-bearing capability, showing considerably higher flexural stresses than the ONYX-CFRP configuration, with PPA-CF-CFRP exhibiting the highest overall flexural response. The PPA-CF-CFRP specimens reached maximum flexural stresses of approximately 149–156 MPa, compared with approximately 106–111 MPa for the ONYX-R-CFRP specimens. Following the maximum stress, both configurations exhibited a pronounced decrease in load-bearing capacity. However, the PPA-CF-CFRP specimens maintained higher stress levels over part of the post-peak strain range, while the ONYX-R-CFRP specimens showed a more progressive reduction in flexural stress. The PPA-CF-CFRP configuration also exhibited a lower strain at failure compared with ONYX-R-CFRP. Coefficients of variation (CV%) for the principal compressive and flexural properties of the investigated sandwich configurations are showed in Table 5.

**Table 5:** Coefficients of variation (CV%) for the principal compressive and flexural properties of the investigated sandwich configurations (*n* = 3).

Configuration	Compression Strength CV (%)	Compression Modulus CV (%)	Max. Flexural Stress CV (%)	Flexural Modulus CV (%)
PPA-CF-CFRP	1.10	2.67	2.74	4.17
ONYX-R-CFRP	5.69	1.44	2.32	1.25
ONYX-CFRP	1.42	12.62	12.60	8.56

### 3.3. DMA Measurements

Dynamic Mechanical Analysis (DMA) was performed in 3-point bending mode at an oscillation frequency of 1 Hz and a maximum force of 18 N to evaluate the thermo-mechanical performance of the developed sandwich structures under dynamic loading. Since all configurations employed identical CFRP face sheets, the differences in the measured viscoelastic response are primarily associated with the investigated 3D-printed core configurations, which differ in constituent material, reinforcement architecture, and manufacturing route.

**Figure 6:**
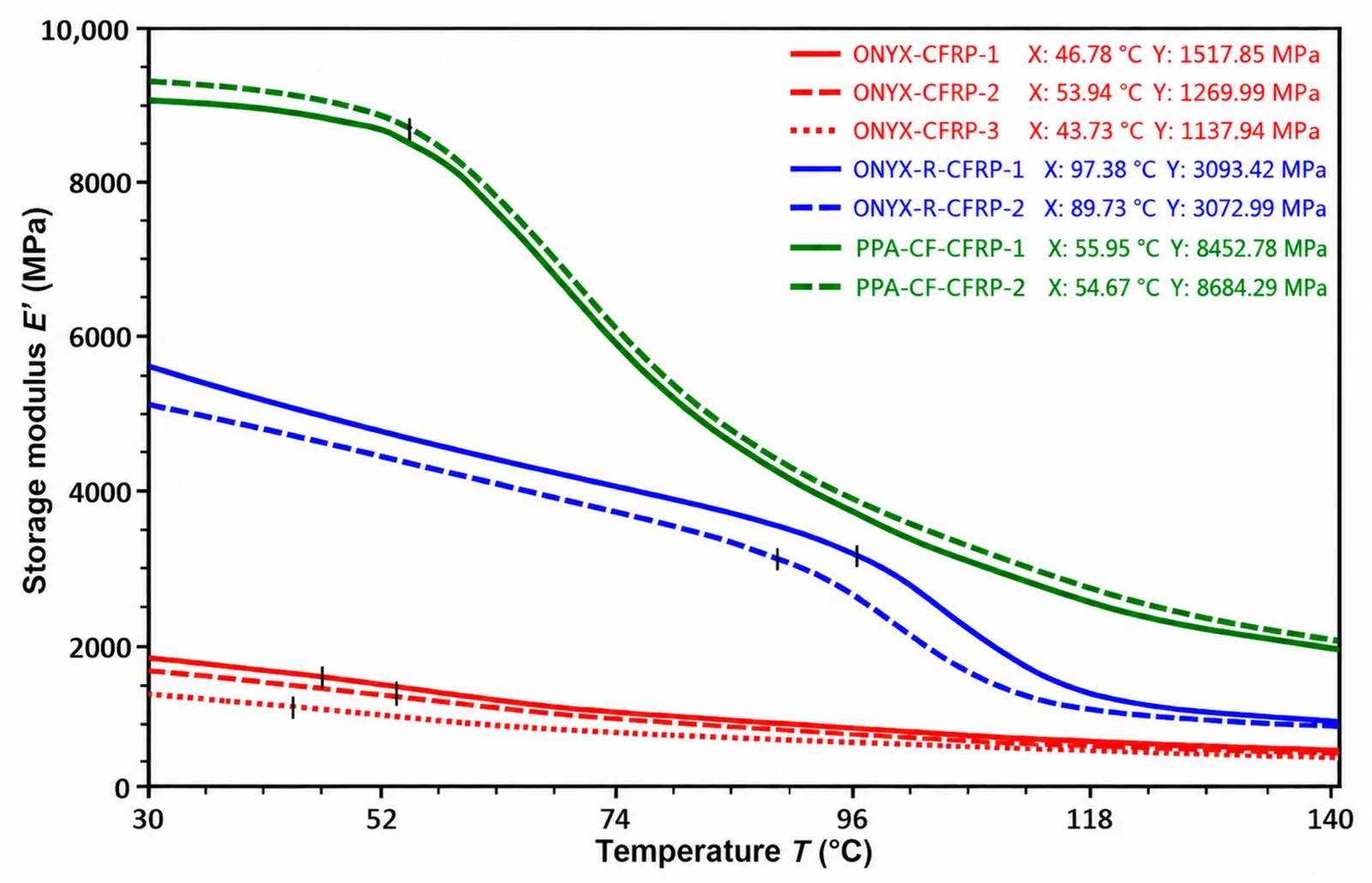
Storage modulus (E”) DMA results.

The storage modulus (E′) curves are presented in Figure 6. At 30 °C, the PPA-CF-CFRP sandwich exhibited the highest storage modulus (approximately 9 GPa), followed by the ONYX-R-CFRP configuration (approximately 5–5.5 GPa), while the ONYX-CFRP sandwich showed the lowest values (approximately 1.2–1.8 GPa). As the temperature increased, all configurations showed a gradual reduction in storage modulus. The PPA-CF sandwich maintained an almost constant stiffness up to approximately 55–60 °C before progressively decreasing while preserving the highest modulus throughout the investigated temperature range. The reinforced ONYX-R-CFRP sandwich showed a gradual decrease followed by a more pronounced reduction above approximately 96 °C, whereas the ONYX-CFRP configuration exhibited a continuous decline over the entire temperature interval.

**Figure 7:**
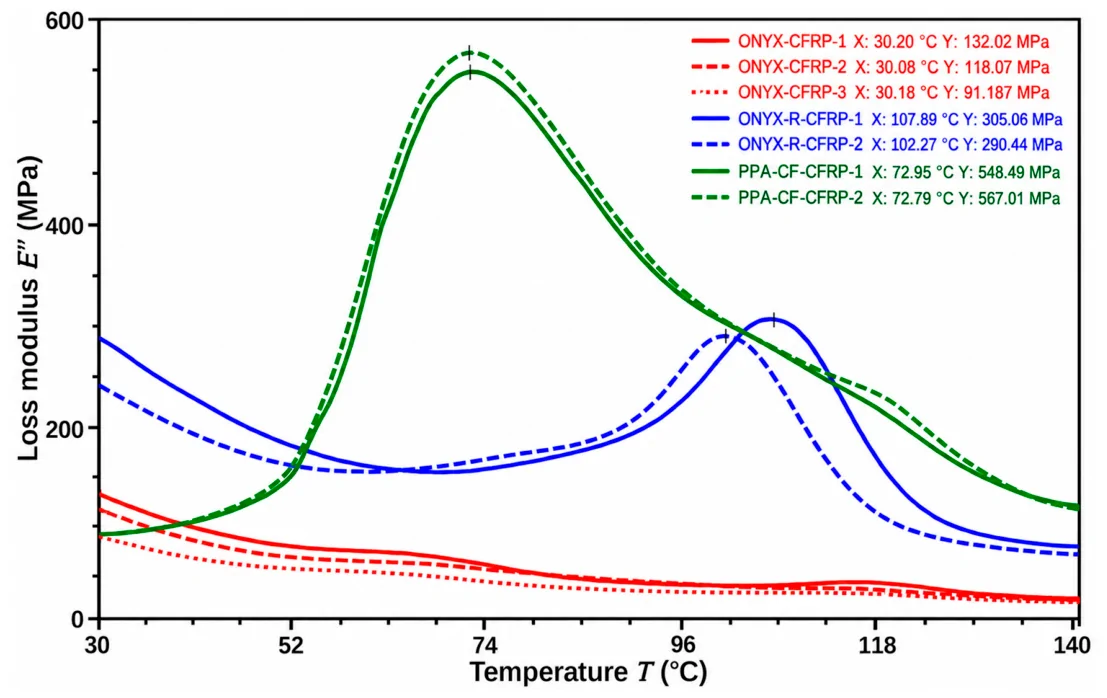
Loss modulus (E”) DMA results.

**Figure 8:**
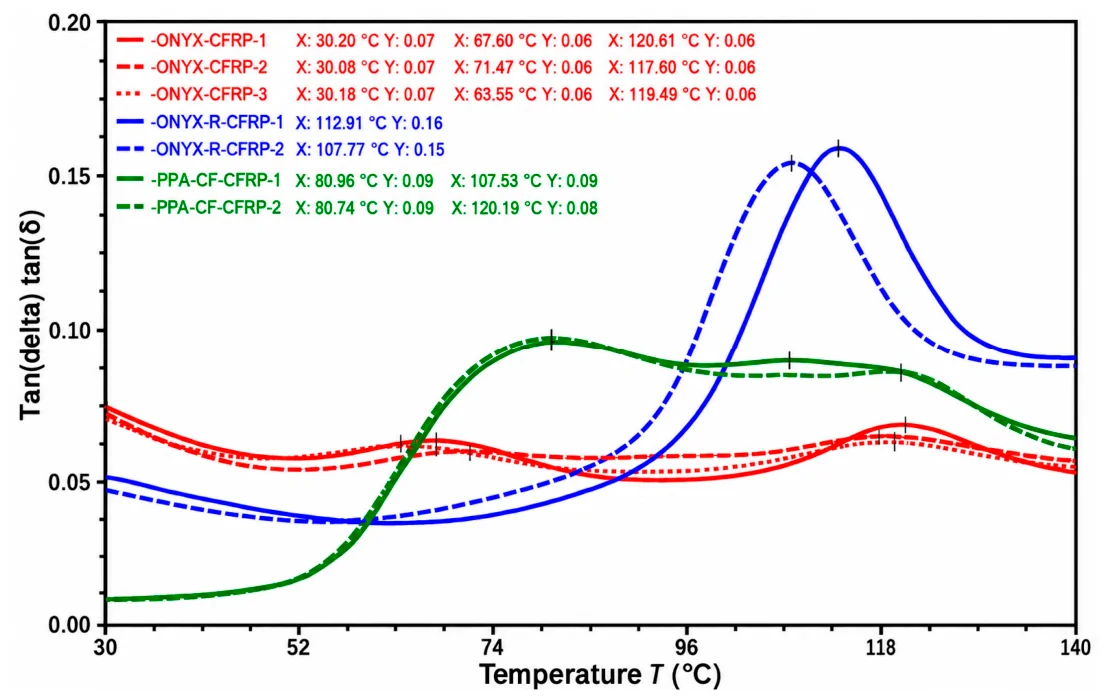
Tan δ DMA results.

The evolution of the loss modulus (E″) with temperature is shown in Figure 7. The PPA-CF sandwich exhibited the highest loss modulus, reaching approximately 550 MPa at around 73 °C. The reinforced ONYX-R-CFRP configuration displayed a lower but well-defined peak of approximately 300 MPa at around 105 °C. In contrast, the ONYX-CFRP sandwich did not exhibit a distinct loss modulus peak, showing only a gradual variation throughout the investigated temperature range. Three specimens were tested for each sandwich configuration. However, the DMA plots present two experimental curves for the PPA-CF-CFRP and ONYX-R-CFRP configurations and three curves for ONYX-CFRP. The reported trends are therefore discussed based on the experimental curves presented in Figure 6, Figure 7 and Figure 8.

The tan δ–temperature curves for the investigated sandwich configurations are presented in Figure 8. Tan δ represents the ratio of loss modulus to storage modulus and provides a measure of the relative dissipative response of the structure. ONYX-R-CFRP exhibited the highest and most clearly defined tan δ peak, reaching approximately 0.16 at 107–113 °C. This peak indicates a pronounced apparent thermomechanical transition occurring at a higher temperature than those observed for the other configurations.

PPA-CF-CFRP exhibited a broader and lower tan δ peak of approximately 0.097 at 80–81 °C, indicating a lower damping factor and an apparent transition occurring at a lower temperature than that of ONYX-R-CFRP. ONYX-CFRP exhibited a comparatively low tan δ response (<0.07), with only weak and broad features over the investigated temperature range, indicating lower structural damping under the applied DMA conditions.

## 4. Discussion

The experimental variability was evaluated using the standard deviation and coefficient of variation (CV) of the measured mechanical properties. The principal compressive and flexural results exhibited different levels of variability among the investigated configurations, with coefficients of variation generally below 6% for the maximum compressive and flexural strengths of PPA-CF-CFRP and ONYX-R-CFRP. However, the ONYX-CFRP configuration exhibited a higher variability in flexural strength (CV = 12.60%). Given the limited number of specimens (*n* = 3 per configuration), these results should be interpreted as descriptive comparisons of the tested sandwich configurations rather than statistically conclusive evidence of intrinsic material superiority. Additional specimens and controlled manufacturing conditions would be required to establish the statistical significance of the observed differences.

The percentage differences reported in this study describe the measured performance of the investigated sandwich configurations under the specified experimental conditions. However, these differences should not be interpreted as the isolated effects of core material or reinforcement type, since the configurations also differ in manufacturing parameters, reinforcement distribution, and apparent core density. Therefore, the observed performance variations reflect the combined influence of material selection, core architecture, and manufacturing conditions. Further investigations using larger sample sizes and more closely controlled manufacturing parameters would be required to establish the individual contributions of these factors.

### 4.1. Compression Test Outcomes

Figure 4 shows distinct flatwise-compression responses for the three sandwich configurations. Since the gyroid topology, nominal core thickness, infill level, and CFRP face sheets were maintained constant across all configurations, the observed differences are primarily associated with the investigated core material systems and their reinforcement architectures. However, because matrix type and reinforcement architecture were not varied independently, their individual contributions cannot be fully decoupled, and the resulting response should be interpreted as the combined effect of these material-related parameters and their interaction within the sandwich structure. Although the CFRP face sheets were directly co-cured onto the prepared thermoplastic cores, the skin–core interfacial bond strength was not independently characterized in the present study. Consequently, the contribution of interfacial adhesion to the observed mechanical response cannot be quantified separately from the effects of core material, reinforcement architecture, and manufacturing parameters. Dedicated interfacial testing will be required to investigate this aspect in future work.

The compression curves of the investigated configurations exhibit regions that can be broadly associated with initial loading, progressive deformation, and a subsequent increase in stress at larger strains. However, the extent and characteristics of these regions differ between configurations, and the underlying local deformation mechanisms were not independently verified. In the initial region, stress increases rapidly as the structure deforms elastically, with PPA-CF-CFRP and ONYX-R-CFRP displaying substantially steeper slopes than ONYX-CFRP. Their mean 0.2% offset yield stresses were 10.17 and 8.59 MPa, respectively (Table 3), whereas ONYX-CFRP developed an early low-stress plateau. In the subsequent plateau region, at approximately 20–45% strain for the reinforced configurations, the observed stress decrease and fluctuations may be associated with progressive deformation and collapse of the gyroid core. The increase in stress at larger strains is consistent with possible densification of the cellular core, although this mechanism was not independently verified. Matrix cracking and local fiber fracture within the printed cell walls may accompany this progressive collapse; however, these microscopic mechanisms cannot be distinguished from the present macroscopic mechanical measurements and were not independently characterized. Thus, the compression response is relevant not only in terms of modulus and strength but also because it reveals how the different printed core systems accommodate deformation and progressive collapse.

**Figure 9:**
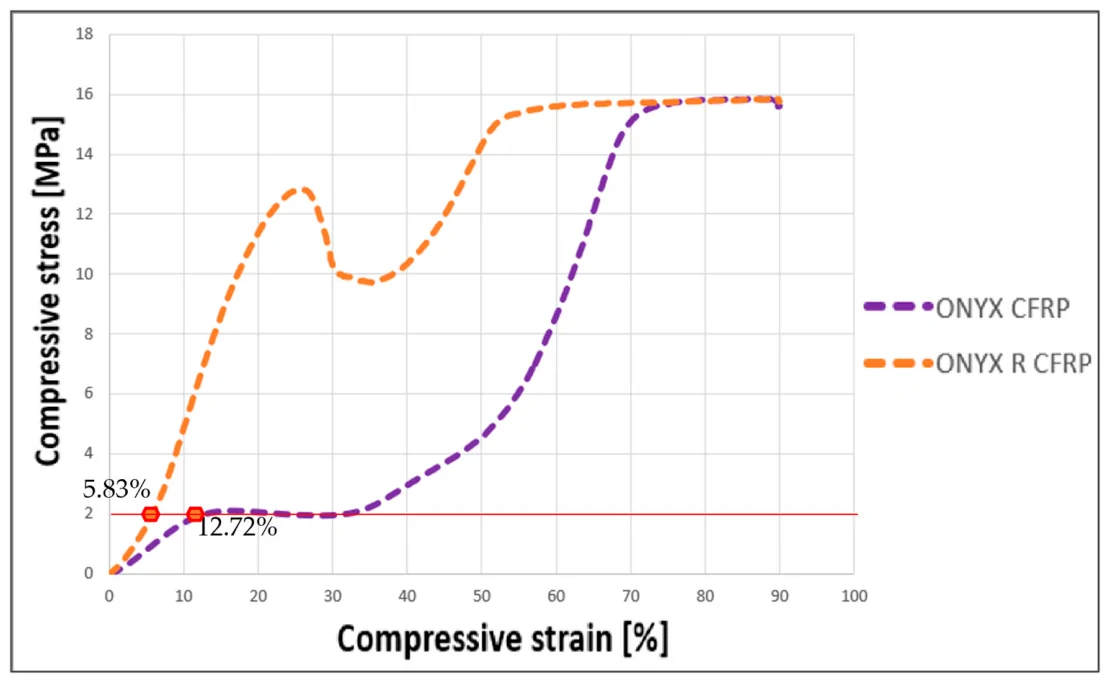
Comparison of the compressive response of ONYX-R-CFRP and ONYX-CFRP at a common stress level of 2 MPa.

The difference in compliance is also evident from the effective compression moduli: ONYX-R-CFRP and PPA-CF-CFRP reach approximately 78 and 87 MPa, respectively, compared with approximately 20 MPa for ONYX-CFRP. Figure 9 illustrates this contrast by comparing ONYX-R-CFRP with ONYX-CFRP at a common stress level. At 2 MPa, the corresponding strains are approximately 5.83% and 12.72%, respectively. Because 2 MPa is close to the mean maximum compression stress of ONYX-CFRP (2.12 MPa), this comparison is not unambiguously pre-yield for both groups and should therefore be interpreted mainly as an illustration of their markedly different compliance. The reinforced ONYX-R core resists deformation more effectively at low and intermediate strain, whereas the more compliant ONYX core reaches its collapse regime much earlier.

A particularly relevant result is the broadly comparable flatwise-compression performance of ONYX-R-CFRP and PPA-CF-CFRP. The mean compression moduli of ONYX-R-CFRP and PPA-CF-CFRP were 77.97 ± 1.12 MPa and 87.25 ± 2.33 MPa, respectively, corresponding to a difference of approximately 12% between the mean values. This difference should be interpreted together with the associated experimental variability rather than solely on the basis of the percentage difference, while their mean compression strengths are 12.84 and 11.85 MPa, respectively (Table 3), despite the different reinforcement architectures. ONYX-R contains continuous carbon-fiber reinforcement, whereas PPA-CF contains only short carbon fibers. This observation suggests that, under flatwise compression, fiber continuity alone does not control the global response. The thermoplastic matrix, local reinforcement distribution, and gyroid cell-wall stability may collectively influence the compressive response, although their individual contributions were not independently quantified. The short-fiber network in PPA-CF may provide effective local constraint against wall deformation and matrix-dominated crushing. Once cell-wall bending, buckling, and progressive collapse become dominant, the potential advantage of continuous fibers aligned along the print path can be less pronounced than under loading modes that exploit reinforcement continuity more directly. Accordingly, the comparable compression strengths of PPA-CF-CFRP and ONYX-R-CFRP suggest that the mechanical response of both configurations is influenced by the deformation and failure of the gyroid core. During testing, the core was observed to fail first; however, the available post-test photographs did not allow the individual local failure mechanisms to be clearly distinguished. Therefore, cell-wall buckling, crushing, and progressive collapse are considered plausible mechanisms rather than independently verified observations.

**Figure 10:**
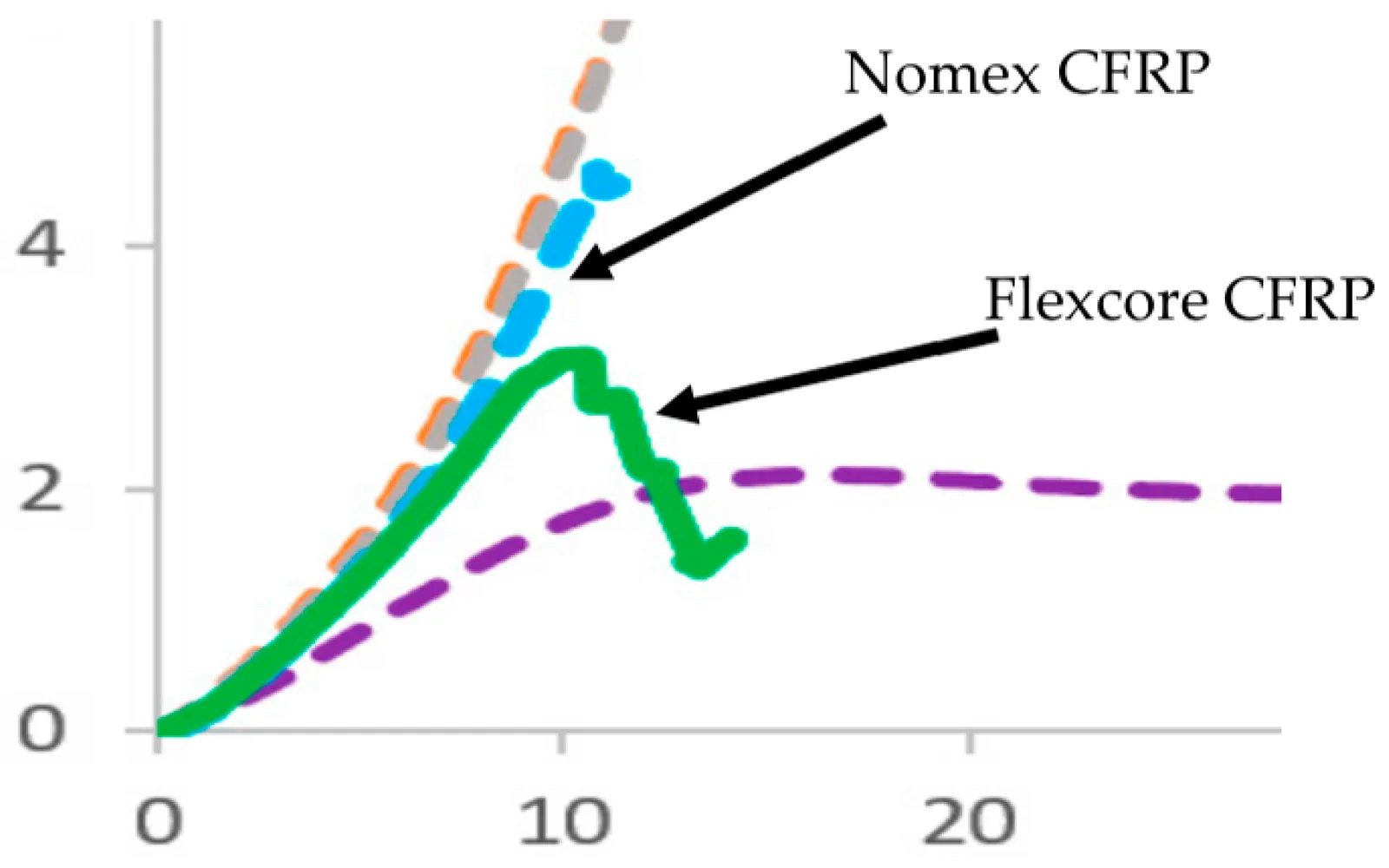
Comparison with Nomex and Flexcore configurations [24].

The comparison with the authors’ previous study [24] provides additional context because conventional Nomex-CFRP and Flexcore-CFRP sandwich structures were investigated under comparable conditions. As shown in Figure 10, the reinforced additively manufactured gyroid-core configurations provide substantially higher flatwise-compression resistance than the conventional cores. This performance gain is, however, accompanied by an increase in specimen mass, so the comparison should consider both absolute strength and mass-related structural efficiency.

For the same specimen dimensions, ONYX-R-CFRP exhibited a mean compressive strength of 12.84 MPa, while PPA-CF-CFRP reached 11.85 MPa, compared with approximately 4.5 MPa for the conventional Nomex-CFRP configuration investigated previously [24]. The density-normalized mechanical properties of the investigated printed-core configurations are presented in Table 6.

**Table 6:** Density-normalized mechanical performance of the investigated CFRP sandwich configurations.

Configuration	Apparent Density–Compression [g/cm^3^]	Specific Compressive Modulus [MPa/(g/cm^3^)]	Specific Compressive Strength [MPa/(g/cm^3^)]	Apparent Density–Bending [g/cm^3^]	Specific Flexural Modulus [MPa/(g/cm^3^)]	Specific Flexural Strength [MPa/(g/cm^3^)]
PPA-CF-CFRP	0.658	132.54	18.00	0.657	16,916.63	231.02
ONYX-R-CFRP	0.691	112.86	18.59	0.688	11,731.02	158.14
ONYX-CFRP	0.583	34.64	3.64	0.587	3130.04	48.79

### 4.2. Three-Point Bending

The three-point bending results differentiate the sandwich configurations more clearly than the flatwise-compression tests. PPA-CF-CFRP reaches the highest flexural stress, approximately 149–156 MPa, followed by ONYX-R-CFRP at approximately 106–111 MPa, whereas ONYX-CFRP reaches only approximately 25–32 MPa but sustains substantially larger deformation (Figure 5 and Table 4). The influence of fiber-reinforcement architecture is particularly evident when comparing ONYX-CFRP and ONYX-R-CFRP. The introduction of continuous carbon-fiber reinforcement in ONYX-R is associated with an increase in mean maximum flexural stress from approximately 28.6 MPa for ONYX-CFRP to 108.7 MPa for ONYX-R-CFRP, indicating increased stiffness and stability of the printed gyroid walls under flexural loading. However, PPA-CF-CFRP, despite containing short carbon-fiber reinforcement, achieved the highest mean maximum flexural stress of approximately 151.7 MPa. These findings suggest that continuous-fiber reinforcement alone is insufficient to explain the differences in flexural performance among the investigated sandwich configurations. Instead, the observed performance results from the combined influence of reinforcement architecture, thermoplastic matrix properties, and the resulting stiffness and stability of the printed gyroid walls. The nearly 40% higher peak flexural stress of PPA-CF-CFRP relative to ONYX-R-CFRP is particularly notable because ONYX-R incorporates continuous carbon-fiber reinforcement. This indicates that the bending response of the complete sandwich cannot be inferred from fiber continuity alone; the effective stiffness of the printed core walls, the compatibility between core and face sheets, and the resulting load-transfer and failure sequence are also important. After the peak load, PPA-CF-CFRP exhibits a comparatively abrupt stress reduction, consistent with its higher stiffness and lower deformation capacity. ONYX-R-CFRP shows a more progressive post-peak decrease and maintains substantial load over a broader strain range, while ONYX-CFRP displays the most compliant and deformation-tolerant response.

The comparison between ONYX-CFRP and ONYX-R-CFRP further illustrates the effect of continuous-fiber reinforcement within an otherwise similar ONYX-based gyroid system. Introducing continuous carbon fibers substantially increases the load-transfer capability and flexural strength of the sandwich, but also changes its failure and post-failure behavior. The unreinforced ONYX core is comparatively compliant, allowing larger global deformation while transferring lower stresses between the CFRP face sheets. The reinforced ONYX-R core carries higher loads but sacrifices part of this deformation tolerance. The overall response therefore highlights an inherent trade-off between flexural strength and deformation capacity. For sandwich optimization, maximizing the strength of the individual constituents is insufficient; stiffness compatibility between the core and CFRP face sheets, the efficiency of load transfer, the local stability of the printed architecture, and the associated failure mechanism must be considered together. This point is particularly important for architected cores, where the global response emerges from the interaction between material properties and cellular geometry rather than from the bulk properties of the thermoplastic alone. From a broader perspective, the flexural performance obtained in the present study can be compared with previously reported sandwich structures incorporating additively manufactured gyroid and other TPMS cores.

The ONYX-R specimens used for compression, three-point bending, and DMA had different nominal continuous-fiber volume fractions and reinforcement layouts, as detailed in Table 1. Therefore, their responses should be interpreted as those of specimen-specific manufactured configurations rather than specimens with identical reinforcement architectures across all three testing methods.

**Figure 11:**
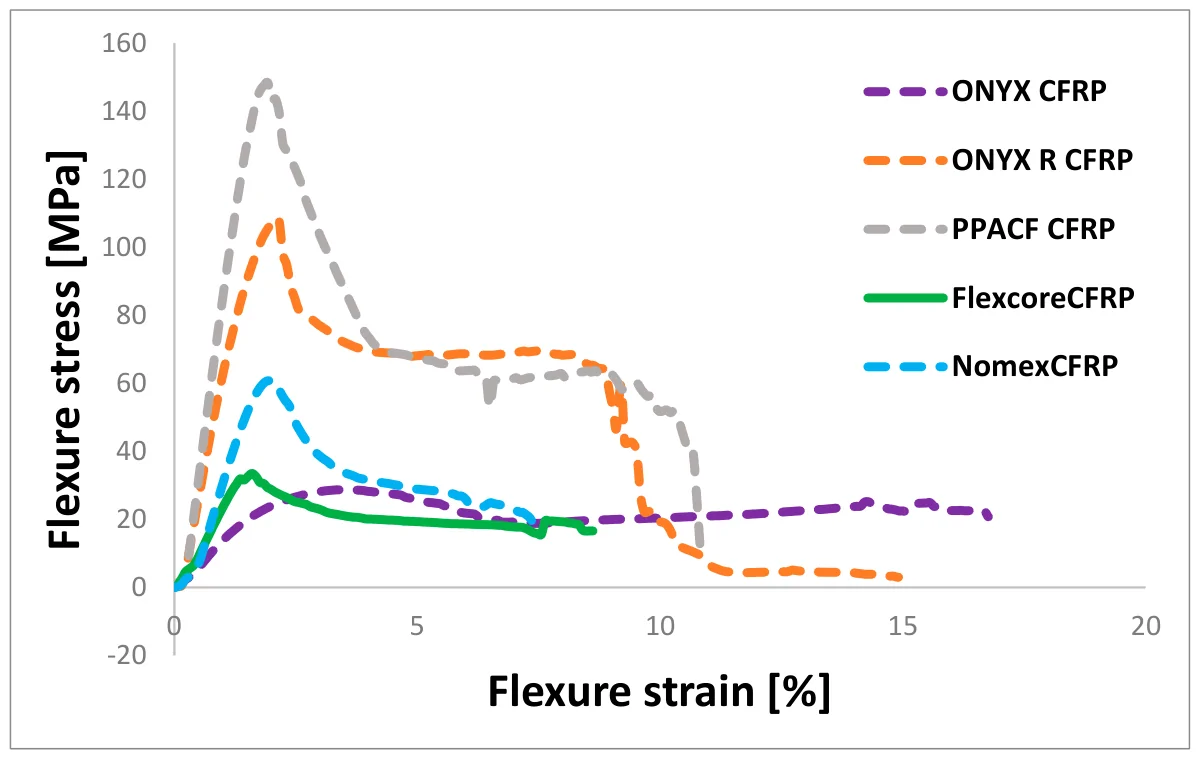
Comparison between printed cores and traditional cores (mean values) [24].

Compared with the conventional sandwich configurations investigated in [24], the printed gyroid cores increase specimen mass, but the reinforced configurations also provide substantially higher flexural performance. The earlier Flexcore-CFRP and Nomex-CFRP specimens had average masses of approximately 8.5 g and peak flexural stresses of about 32 and 60 MPa, respectively. In the present study, ONYX-R-CFRP and PPA-CF-CFRP reached approximately 108.7 and 151.7 MPa, with reported specimen masses of 16.20 and 15.22 g. PPA-CF-CFRP therefore combines the highest peak stress with a lower mass than ONYX-R-CFRP, indicating a favorable strength-to-mass response among the printed configurations. Figure 11 illustrates this balance between performance gain and mass penalty. For weight-sensitive applications, these results suggest that the selection of an additively manufactured core should be based on the required combination of strength, deformation tolerance, and mass rather than on maximum flexural stress alone.

When normalized by the apparent sandwich density, PPA-CF-CFRP exhibited the highest specific compressive stiffness, reaching approximately 132.54 MPa/(g/cm^3^), compared with 112.86 MPa/(g/cm^3^) for ONYX-R-CFRP and only 34.64 MPa/(g/cm^3^) for ONYX-CFRP (Table 6). In terms of specific compressive strength, PPA-CF-CFRP and ONYX-R-CFRP exhibited very similar values of 18.00 and 18.59 MPa/(g/cm^3^), respectively, whereas ONYX-CFRP reached only 3.64 MPa/(g/cm^3^). Therefore, although ONYX-R-CFRP achieved the highest absolute compressive strength, its greater apparent density reduced this advantage after mass normalization. Conversely, PPA-CF-CFRP provided the best stiffness-to-density balance among the investigated configurations.

Apparent sandwich density was calculated from the average specimen mass and measured specimen dimensions. Specific stiffness and specific strength were calculated by normalizing the corresponding modulus and strength values by the apparent sandwich density.

The lightweight-performance advantage of PPA-CF-CFRP becomes even more evident under flexural loading. After normalization by the apparent sandwich density, PPA-CF-CFRP reached a specific flexural strength of approximately 231.02 MPa/(g/cm^3^), compared with 158.14 MPa/(g/cm^3^) for ONYX-R-CFRP and 48.79 MPa/(g/cm^3^) for ONYX-CFRP. Similarly, its specific flexural modulus reached approximately 16,916.63 MPa/(g/cm^3^), exceeding ONYX-R-CFRP (11,731.02 MPa/(g/cm^3^)) and ONYX-CFRP (3130.04 MPa/(g/cm^3^)). These results demonstrate that the superior absolute flexural performance of the PPA-CF core is retained after density normalization and is therefore not simply a consequence of increased structural mass.

### 4.3. DMA Test Outcome

DMA revealed pronounced differences in the temperature-dependent response of the investigated sandwich configurations. Because all configurations employed identical CFRP face sheets, these differences primarily reflect the combined effects of the 3D-printed core material, reinforcement architecture, gyroid geometry, skin–core interaction, and the resulting global structural response. The DMA data should therefore be interpreted at the sandwich level rather than as direct measurements of the intrinsic viscoelastic properties of the individual core polymers.

The temperature-dependent storage-modulus response shows that PPA-CF-CFRP provides the highest initial stiffness, approximately 9 GPa at 30 °C, followed by ONYX-R-CFRP at approximately 5–5.5 GPa and ONYX-CFRP at approximately 1.5–1.8 GPa. More importantly for service-temperature behavior, the configurations do not lose stiffness in the same temperature range. PPA-CF-CFRP begins a pronounced reduction in E’ at approximately 50–55 °C, whereas the stronger decrease in ONYX-R-CFRP is shifted toward approximately 95 °C. These temperatures should be regarded as features of the global thermomechanical response of the sandwich structures, not as direct glass-transition temperatures of the constituent matrices, because matrix chemistry, reinforcement architecture, printed geometry, interface behavior, and manufacturing history can all contribute to the measured response. The loss-modulus curves reinforce this distinction. PPA-CF-CFRP exhibits the largest E’’ peak, approximately 550–570 MPa at 73–74 °C, whereas ONYX-R-CFRP shows a lower and broader peak of approximately 300 MPa at 102–108 °C. The shift in the ONYX-R-CFRP loss-modulus maximum toward higher temperature is consistent with the delayed reduction observed in its storage modulus. ONYX-CFRP, in contrast, shows a relatively low and weakly varying loss modulus across the investigated temperature range, indicating a lower dissipative response under the applied DMA conditions. Taken together, E’ and E’’ therefore indicate different apparent thermomechanical transition windows: PPA-CF-CFRP combines the greatest low-temperature stiffness with an earlier transition region, whereas ONYX-R-CFRP retains its stiffness response to a higher temperature and exhibits a delayed dissipative maximum. The tan δ curves provide a complementary measure of this behavior. ONYX-R-CFRP exhibits the highest damping peak, approximately 0.16 at 107–113 °C, PPA-CF-CFRP shows a broader and lower maximum of approximately 0.097 at 80–81 °C, and ONYX-CFRP remains below approximately 0.07 over most of the investigated range. Thus, the configuration with the highest room-temperature stiffness is not the configuration with the highest-temperature damping maximum. This distinction is relevant for design because it shows that core selection can be used to tailor stiffness retention and damping independently to some extent, depending on the expected operating-temperature range.

Comparison with the conventional Nomex-CFRP and Flexcore-CFRP sandwich structures investigated previously [24] further clarifies the magnitude of the stiffness increase associated with the reinforced printed-core configurations. The comparison was performed on a consistent experimental basis, as the specimens were manufactured and tested by the same research team using the same HexPly^®®^ M49 CFRP face-sheet material, equivalent face-sheet configuration and thickness, an approximately 6 mm core thickness, and comparable specimen dimensions (approximately 60 × 12 × 6 mm). DMA measurements were performed using the same TA Instruments DMA 850 equipment, in three-point bending mode, at an oscillation frequency of 1 Hz and a maximum force of 18 N, following ASTM D7028-07(2015), under equivalent temperature-sweep conditions. At 30 °C, PPA-CF-CFRP reaches approximately 9 GPa, corresponding to increases of about 250% relative to Nomex-CFRP and 500% relative to Flexcore-CFRP, while ONYX-R-CFRP reaches approximately 5–5.5 GPa, corresponding to increases of about 135% and 270%, respectively. ONYX-CFRP remains within the approximate storage-modulus range of the conventional configurations. The higher stiffness of PPA-CF-CFRP and ONYX-R-CFRP is accompanied by a substantial mass penalty, emphasizing that the advantage of printed cores should be evaluated in terms of stiffness-to-mass efficiency rather than stiffness alone. Their higher storage moduli are consistent with reduced global compliance and a stronger contribution of the core to the flexural response of the sandwich structure. Nevertheless, these values represent the effective thermo-mechanical response of the complete sandwich structures rather than the intrinsic modulus of the core materials. Furthermore, the present measurements do not isolate the contribution of the skin–core interface; therefore, the observed improvement cannot be assigned specifically to reduced interfacial mismatch. In addition to increasing stiffness, the printed-core configurations shift the temperature range of the dominant viscoelastic response. Consequently, additive manufacturing provides a route not only to geometric customization but also to tailoring the balance between mechanical stiffness, damping, and temperature-dependent performance through the selected core material and reinforcement architecture.

## 5. Conclusions

The mechanical and thermomechanical behavior of CFRP sandwich structures incorporating additively manufactured gyroid cores was strongly dependent on the investigated core configuration. The main findings can be summarized as follows:

1. PPA-CF-CFRP exhibited the highest flexural response, with an average maximum flexural stress of approximately 151.7 MPa, compared with 108.7 MPa for ONYX-R-CFRP and 28.6 MPa for ONYX-CFRP. Conversely, ONYX-CFRP exhibited the highest deformation capacity, with a flexural strain at break of approximately 21%. These results indicate a trade-off between load-bearing capability and deformation capacity among the investigated configurations.
2. In flatwise compression, PPA-CF-CFRP exhibited the highest apparent compression modulus (~87 MPa), followed by ONYX-R-CFRP (~78 MPa) and ONYX-CFRP (~20 MPa). Despite their different reinforcement architectures, PPA-CF-CFRP and ONYX-R-CFRP showed comparable mean compression strengths of 11.85 and 12.84 MPa, respectively. The observed differences may reflect the combined influence of core material, reinforcement architecture, and as-built structural characteristics; however, the individual contributions of these factors were not independently determined.
3. DMA revealed distinct temperature-dependent stiffness and damping responses among the three configurations. PPA-CF-CFRP exhibited the highest storage modulus at 30 °C (~9 GPa), whereas ONYX-R-CFRP showed the highest damping factor (tan δ ≈ 0.16 at 107–113 °C) and a higher-temperature apparent thermomechanical transition. ONYX-CFRP exhibited the lowest storage modulus and the weakest damping response.
4. Compared with the conventional Nomex-CFRP and Flexcore-CFRP sandwich systems investigated previously [24], the reinforced additively manufactured gyroid-core configurations provided substantially higher flexural, compression, and dynamic-stiffness responses, although at the expense of increased structural mass. The results therefore indicate that gyroid-core selection should be based on the required balance among stiffness, strength, deformation capacity, damping response, temperature-dependent behavior, and mass. Further optimization of relative density, reinforcement distribution, and lattice architecture is required to improve the overall structural efficiency of these systems.

The conclusions presented above are based on the experimentally measured mechanical and thermomechanical responses of the investigated sandwich configurations. While the results suggest that core material, reinforcement architecture, and manufacturing parameters collectively influence structural performance, the individual contributions of these factors could not be isolated. Similarly, specific local damage mechanisms and skin–core interfacial effects were not independently characterized. Therefore, the proposed explanations should be regarded as interpretations of the observed behavior rather than experimentally confirmed mechanisms. Considering the limited sample size (*n* = 3 per configuration), further investigations involving additional specimens, controlled manufacturing parameters, and detailed failure characterization are required to validate these interpretations.

## Figures and Tables

**Table 1:** 3D-Printed core features.

Feature	Core Type				
Material	PPA-CF	ONYX	Carbon-fiber-reinforced ONYX		
Printer	Prusa CoreOneL	Markforged X7	Markforged X7		
Slicer	Prusa Slicer	Eiger	Eiger		
Layer Height	0.15 mm	0.125 mm	0.125 mm		
Nozzle Diameter	0.4 mm	0.4 mm	0.4 mm		
Nozzle temperature	290 °C	275 °C	275 °C		
Bed temperature	50 °C	—	—		
Number of walls	1	1	1		
Bottom layers	2	1	1		
Top Layers	2	1	1		
Infill	Gyroid 28%	Gyroid 28%	Gyroid 28%		
Fiber Stripe count	—	—	1 stripe per 6 mm height		
Layers per stripe	—	—	4		
Continuous fiber volume fraction	—	—	Compression	3-Point Bending	DMA
			~8%	~9.3%	~7.4%

**Table 2:** Experimentally measured core masses and calculated apparent densities of additively manufactured gyroid cores for different specimen configurations.

Material	DMA Mass (g)	DMA (g/cm^3^)	Compression Mass (g)	Compression (g/cm^3^)	Bending Mass (g)	Bending (g/cm^3^)
PPA-CF	2.55	0.590	10.96	0.507	11.08	0.513
ONYX-R	2.41	0.558	12.64	0.585	12.67	0.587
ONYX	2.21	0.512	10.67	0.494	10.31	0.477

**Table 3:** Flatwise compression properties of the investigated sandwich configurations (*n* = 3). Values are reported as mean ± standard deviation (SD).

Sandwich Configuration	Sample No.	Compression Modulus (MPa)		Yield Strength (0.2%) [MPa]		Compression Strength [MPa]	
CFRP/PPA-CF	1	89.84	87.25 ± 2.33 *	10.50	10.17 ± 0.29 *	11.97	11.85 ± 0.13 *
	2	85.33		9.95		11.72	
	3	86.57		10.05		11.85	
CFRP/ONYX-R	1	78.13	77.97 ± 1.12 *	8.16	8.59 ± 0.66 *	12.36	12.84 ± 0.73 *
	2	76.77		8.26		12.48	
	3	79.00		9.35		13.68	
CFRP/ONYX	1	18.00	20.20 ± 2.55 *	1.65	1.55 ± 0.20 *	2.14	2.12 ± 0.03 *
	2	19.61		1.32		2.12	
	3	22.99		1.69		2.09	

* mean values with standard deviation.

**Table 4:** Three-point bending results for individual sandwich specimens.

Sandwich Configuration/ Sample No.		Max. Flexural Stress [MPa]	Flexural Stress at Break [MPa]	Flex Strain at Break [%]	Energy at Break [J]	Modulus [MPa]
CFRP/PPA-CF	1	156.50	37.50	6.49	8.41	10,594.06
	2	148.84	11.54	10.83	11.08	11,488.50
	3	149.86	29.15	10.77	12.73	11,251.34
	Mean ± SD	151.74 ± 4.16	26.06 ± 13.25	9.36 ± 2.49	10.74 ± 2.18	11,111.30 ± 463.37
CFRP/ONYX-R	1	106.21	17.19	9.07	9.29	7993.43
	2	108.74	4.20	12.62	10.70	8024.11
	3	111.26	3.00	14.91	10.86	8181.43
	Mean ± SD	108.74 ± 2.52	8.13 ± 7.87	12.20 ± 2.94	10.28 ± 0.86	8066.32 ± 100.86
CFRP/ONYX	1	32.33	15.04	20.52	5.51	2019.09
	2	28.46	16.75	20.76	5.57	1752.26
	3	25.12	14.13	21.70	4.33	1741.26
	Mean ± SD	28.64 ± 3.61	15.31 ± 1.33	20.99 ± 0.62	5.14 ± 0.70	1837.54 ± 157.33

## Data Availability

The original contributions presented in this study are included in the article. Further inquiries can be directed to the corresponding author.
